# Microneedles for Chronic Refractory Wounds: A Review of Dynamic Regulation Strategies and Therapeutic Applications

**DOI:** 10.3390/ijms27198520

**Published:** 2026-09-24

**Authors:** Quanying Liu, Yang Xu, Wenfeng Li, Chunmeng Shi

**Affiliations:** State Key Laboratory of Trauma and Chemical Poisoning, Institute of Rocket Force Medicine, Third Military Medical University (Army Medical University), Gaotanyan Road Street, Shapingba District, Chongqing 400038, China; l_ying@tmmu.edu.cn (Q.L.); xuyang217327@tmmu.edu.cn (Y.X.);

**Keywords:** chronic refractory wounds, microneedles, dynamic regulation, regenerative microenvironment, wound healing

## Abstract

Chronic refractory wounds (CRWs), characterized by delayed healing due to disrupted phases of hemostasis, inflammation, proliferation, and remodeling, pose significant clinical challenges with diverse etiologies such as diabetic ulcers and radiation-induced ulcers. Traditional therapies like debridement and skin flap transplantation face limitations. Microneedle technology has emerged as a promising approach, enabling painless transdermal drug delivery to the dermis while enhancing bioavailability and enabling microenvironment-responsive interventions. In this context, this review highlights advancements in microneedle design, emphasizing material selection and structural innovations tailored for CRW management, and systematically elucidates the mechanisms of action and applications of microneedles for phased regulation of CRW healing in response to the pathologic characteristics of wounds—hemostatic, inflammation regulation, angiogenesis and remodeling stage—in terms of prevention, intervention, and wound monitoring, thereby providing significant reference value for the design and clinical application of microneedles in chronic refractory wounds.

## 1. Introduction

Chronic refractory wounds (CRWs) fail to heal within the expected time and impose a growing clinical and socioeconomic burden [1,2,3]. Normal wound repair is commonly described as four overlapping phases: (1) initiation of the coagulation mechanism for hemostasis; (2) cytokine release and leukocyte infiltration causing an inflammatory response; (3) proliferation, including angiogenesis, re-epithelialization, and extracellular matrix (ECM) deposition; and (4) remodeling of the ECM over several weeks to months [4,5]. Disruption of the timing, magnitude, or resolution of these processes can produce a chronic wound. Clinically important CRWs include diabetic ulcers, pressure ulcers, traumatic ulcers, and radiation-induced skin injury (RISI), although their etiologies and dominant pathological mechanisms differ [6,7,8].

The traditional surgical treatments for CRWs mainly include debridement and implantation, negative-pressure wound therapy, and skin flap transplantation [9,10]. However, these approaches have limitations, including insufficient donor tissue and the risk of flap necrosis [11,12]. Meanwhile, traditional dressings and local medications make it difficult to penetrate the dense necrotic tissue, resulting in low bioavailability and insufficient targeting, in which the therapeutic drugs cannot effectively reach the deep tissue [13,14]. In contrast, MNs physically traverse superficial barriers and deliver cargo into viable tissue while reducing systemic exposure. Their small dimensions and patch-based administration can also improve tolerability and ease of use. In addition, responsive materials and integrated sensors may enable treatment to be adjusted to local pH, reactive oxygen species (ROS), temperature, enzymes, or biochemical markers [15,16,17,18].

Previous reviews have examined MN materials, fabrication, drug delivery, sensing, or wound applications [15,19,20,21,22,23]. However, there is a lack of comprehensive discussion on the current advances and challenges in the use of microneedles for the prevention, monitoring, and regeneration of CRWs. Therefore, we conducted a detailed review focusing on the consideration and design of microneedles to assist wound healing and regeneration in various CRWs. Here, this review presents the classification and design strategies of microneedle systems in terms of material selection and structural design. Subsequently, in order to better understand the role of microneedles in CRWs, this review introduces the mechanisms of action and applications of microneedles for phased regulation of CRW healing in response to the pathologic characteristics of wounds, in terms of prevention, intervention, and wound monitoring. Evidence is interpreted according to model relevance: diabetic, infected, and radiation-induced wound studies are treated as direct CRW evidence, whereas acute incisional wounds, hepatic hemorrhage, oral ulcers, and pathological scars are identified as mechanistic or proof-of-concept models. Findings from the latter models should not be interpreted as clinical efficacy in CRWs without model-specific validation. In conclusion, a thorough examination of the constraints and prospective developments regarding the application of microneedles in the management of chronic wound conditions is presented and discussed.

## 2. Classification and Design Strategy of Microneedles

Microneedle patches (MNs) represent a rapidly advancing transdermal delivery platform with broad applicability across medical fields. These devices, currently under intensive development, have demonstrated utility in cosmetic enhancement, vaccine administration, diagnostic monitoring, and therapeutic interventions such as diabetes management [24]. Typical MN configurations feature precise microarchitectures, with needle dimensions generally ranging from 150 to 1500 μm in vertical height, 50 to 500 μm in base diameter, and 1 to 25 μm in tip radius of curvature [25,26]. Geometry, density, material strength, and insertion force collectively determine penetration and delivery performance.

### 2.1. Structural Architectures and Delivery Mechanisms

MN systems should first be classified by how they create a transport pathway and where the payload resides. Different microneedle structures have been designed to precisely control the distribution and release of multiple drugs in microneedles benefiting from the current advanced manufacturing technologies [16,27]. Based on the structural properties, the major delivery architectures are solid, coated, hollow, dissolving, and hydrogel-forming MNs; layered and core–shell arrangements are cross-cutting architectures that can be incorporated into several of these types.

Solid microneedles work by penetrating the skin barrier to form micro-channels in the skin, which allow for passive diffusion of drug agents through the channels into the dermis. Following MN removal, transdermal administration involves applying a drug-loaded patch to the treated area, where passive diffusion of drug molecules through these microchannels facilitates gradual permeation into deep dermal tissues [25,28]. Their advantages include simple fabrication, high insertion strength, and broad compatibility with formulations. Limitations include a two-step procedure, variable pore lifetime, and drug delivery that depends on passive diffusion rather than an MN-contained dose.

Coated microneedles are another type of solid microneedles in which the outer surface of the solid microneedle is covered with a drug coating, and when the microneedle is inserted into the skin, the drug coated on the microneedle is released directly [29]. Due to the small drug loading capacity of coated microneedles, they are mostly used for vaccinations where only a small amount of antigen is needed to induce an immune response [30]. Coated MNs provide accurate surface localization and are well suited to potent, low-dose cargos such as vaccines, but coating thickness and needle surface area restrict loading capacity, and incomplete coating transfer can increase dose variability. There are also coated microneedles designed with a core–shell structure; microneedles with inner and outer layers are designed with different materials to achieve different controlled release rates on both sides [31,32], increase the drug delivery dose, and improve the efficiency of drug delivery. The introduction of some environmentally responsive materials can also realize controlled release [33,34,35]. These microneedles can precisely control the amount of drug administered, avoiding the residual risk of needle breakage in traditional injections, while integrating therapeutic functions such as antimicrobial and enzymatic activity and achieving the synergistic effects of drug release and wound microenvironmental response, which still makes them irreplaceable in areas such as chronic wound management.

Hollow microneedles contain an internal cavity structure which can be used to load drugs down the concentration gradient by pressure driven into the body to achieve drug delivery [36,37]. A lumen and tip or side opening deliver liquid formulations by means of externally applied pressure, a pump, capillary action, or a concentration gradient. Generally, non-degradable materials are used to make a shell with certain mechanical strength, and drugs are made into nanoparticles [38], liposomes, etc., loaded in the center cavity and then released from the tip of the needle or the holes in the side wall of the shell under the effect of pressure after piercing the skin. In addition to being able to accommodate higher doses of drugs, hollow microneedles have the advantage of rapid delivery of loaded substances and enhanced transdermal delivery of nanocarriers [39,40]. They can deliver larger, titratable doses and accommodate suspensions or nanocarriers, but they require mechanically stable shells and may clog in protein-rich exudate or tissue debris; leakage and flow resistance also complicate dose control.

Dissolving MNs operate through a “poke-and-release” mechanism, in which the payload is incorporated into a water-soluble or biodegradable needle matrix that dissolves after insertion, leaving no sharp waste. This design enables single-step administration and allows cargo to be concentrated in the needle tips. However, loading capacity is limited by needle volume, rapid dissolution may cause burst release, and high payload concentrations can compromise mechanical strength. In contrast, hydrogel-forming MNs consist of cross-linked needles that absorb interstitial fluid and swell after insertion, creating aqueous channels through which drugs diffuse from the swollen matrix or an attached reservoir. As an ECM-like material with high water content, hydrogel-like polymers are suitable for loading water-soluble drugs and proteins, nanoparticles, nucleic acids, EVs and cells. Due to their tunable polymer network density, hydrogels can also be designed to control drug release based on drug size. Hydrogel microneedles are made of soluble cross-linked polymers, and after piercing the skin, the polymer absorbs tissue fluid and undergoes swelling, and the drug molecules are released through the swelling’s loosened pores. Common hydrogel microneedles are generally made of temperature-responsive poly(N-isopropylacrylamide) (PNIPAM) [41,42], gelatine methacrylate (GelMA) [43,44], poly(vinyl alcohol) (PVA) [45] and poly(ethyleneglycol diacrylate) (PEGDA) [46,47], which all have good biocompatibility.

Layered, stacked, and core–shell MNs spatially separate cargos or materials to preset their order and rate of release. Stacked structure microneedles are a common class of shaped microneedles [45]. Unlike core–shell structures with sequential drug release, stacked structure microneedles stack multiple matrix layers on a single tip. By regulating the degradation rate of different layers, programmed release of multiple drugs is achieved [48]. In addition, this stacked structure will better promote tissue adhesion of the microneedle [49]. Bioinspired microneedles are a new microneedle technology developed by mimicking the structural and functional properties of biological systems. By mimicking nature-based designs, the function of the microneedle is altered to achieve a specific desired result. The design is inspired by biological structures in nature that have efficient penetration or material delivery capabilities, and the mechanical properties of microneedles, tissue interaction patterns and drug delivery efficiency are optimized through bionic engineering strategies [50]. For example, microneedles with unique bionic serrated structures have been shown to have better hemostatic properties [45,51,52], and microneedles inspired by octopus suction cups have better tissue adhesion [53]. They can increase functional complexity and reduce incompatibility between cargos, but the programmed sequence is determined mainly by layer thickness, matrix degradation, and diffusion. Without a sensor and an active control element, such devices should be described as programmed-release systems rather than feedback-controlled systems.

### 2.2. Materials Used for Microneedle

Material classification is independent of delivery architecture. MNs may be fabricated from inorganic materials (including silicon and ceramics), metals, natural or synthetic polymers, and composites [16]. Material selection determines stiffness, fracture behavior, degradation, manufacturability, cargo compatibility, and the achievable release profile.

Different materials are selected for microneedle fabrication according to application-specific mechanical and degradation requirements [16]. In general, they can be categorized into ceramic microneedles, metal microneedles, polymer microneedles and composite microneedles.

Ceramic microneedles are a class of microneedles made of inorganic non-metallic materials produced by a molding process; they have relatively high stiffness (more than 10 GPa) and slow degradation in vivo. Within the MN literature, silicon-based devices are commonly discussed alongside ceramic systems because both offer high stiffness and are compatible with microfabrication. Representative ceramics include alumina [54] and zirconia [55], which provide high hardness and chemical stability, and porous calcium–phosphate coatings [56,57], which can immobilize cargo and access the epidermis. Their stiffness supports reliable penetration, but brittleness, limited intrinsic loading capacity, high-temperature processing, and difficult degradation control require careful design.

Metallic microneedles, often made of stainless steel, titanium, nickel, and palladium, typically have a fairly good combination of mechanical properties, including high toughness, high strength, and high hardness, which can protect MNs from mechanical failure [58]. In addition, metal MNs are relatively inexpensive to fabricate compared to silicon MNs, and can be manufactured using a variety of methods such as electroplating, photochemical etching, and laser cutting [59]. Although metals such as stainless steel and titanium have been safely used in the medical field for decades, the safety of other metals requires further study.

Polymers are generally highly ductile, so polymer MNs will not fracture brittlely when inserted into skin or other tissues. Polymer MNs provide a broad range of mechanical and degradation properties and can be tailored by molecular weight, concentration, crystallinity, cross-link density, and charge [60,61]. Biodegradable aliphatic polyesters such as poly(lactic acid) (PLA) [62,63,64] and polycaprolactone (PCL) [62,65], which are used in regulated biomedical products, have been fabricated into MNs. PLA generally provides relatively high stiffness, whereas PCL is more flexible and degrades more slowly; these properties create a trade-off between insertion stability and timely cargo release. Natural polymers and hydrogels can accommodate proteins, nanoparticles, nucleic acids, extracellular vesicles, and cells, but their water content and processing conditions can reduce mechanical strength.

### 2.3. Functional and Formulation Design

Functional design overlays the primary delivery architecture. Bioinspired serrations [52], suction cup-like surfaces [53], and pagoda [45] or kirigami geometries [66] can improve penetration, adhesion, fixation, or hemostasis. Core–shell and stacked domains can program a release sequence, while pH-, ROS-, temperature-, radiation-, enzyme-, or light-responsive components can change release in response to a signal. Sensing modules form a separate functional layer. Consequently, “shaped” MNs are functional geometries rather than a category equivalent to solid or hollow MNs, and one device may occupy several design categories.

Cryo-microneedles are likewise a formulation and fabrication strategy rather than a material class. A cryoprotected aqueous formulation is frozen into an MN geometry, inserted while mechanically rigid, detached, and then thawed to release its cargo. By controlling the degree of cell loading and the length of the microneedle, the effective dosage of cell delivery and the depth of delivery can be accurately controlled [67,68]. Therefore, various types of therapeutic cells can be packaged in cryo-microneedles. For example, cryo-microneedles can deliver melanocytes for vitiligo treatment or stem cells to promote skin regeneration. Cryo-microneedles can also be used to deliver probiotic bacteria or bacteriophages for the treatment of topical infections [69]. Chang et al. loaded tumor antigen ovalbumin (OVA)-pulsed dendritic cells (DCs) into cryo-microneedles [70]. In contrast to conventional injection methods for DC delivery (e.g., intravenous and subcutaneous injections), this cryo-microneedle strategy can pierce the skin, deliver loaded living cells and easily enable intradermal delivery and immunization of DCs. These studies establish delivery feasibility, but they do not by themselves establish the efficacy in CRWs unless the intended chronic wound model is tested.

## 3. Dynamic Regulation of Microneedles in Chronic Wound Repair

Wound healing is a highly coordinated process involving hemostasis, inflammation, angiogenesis, cell proliferation and connective tissue formation, wound contraction, and tissue remodeling. These interconnected and partially overlapping stages normally progress in an orderly manner under precise physiological regulation [71]. Chronic wounds, however, fail to restore anatomical and functional integrity through this timely and coordinated repair process. Their pathogenesis is characterized by a dynamic, multifactorial imbalance, with persistent inflammation, impaired angiogenesis, and abnormal extracellular matrix (ECM) remodeling representing the principal pathological features [9,72,73]. These mechanisms are interrelated and together they lead to stagnant wound repair, forming a closed loop pathology characterized by “persistent inflammation–ischemia and hypoxia–matrix destruction–infection and drug resistance” (Figure 1).

Consequently, chronic wounds often exhibit an exceptionally hostile microenvironment characterized by inadequate vascularization, severe hypoxia, elevated levels of pro-inflammatory cytokines, infection, necrotic tissue, eschar, persistent exudation, ulceration, and biofilm formation. These conditions reduce drug retention and impede the penetration of topically administered agents into the wound bed. Compared with conventional dressings and intravenous administration, microneedles may help address these limitations by bypassing physical barriers and releasing drugs in a rapid or sustained manner, thereby increasing therapeutic availability to the wound bed. At the same time, through the rational design of the microneedle, it can dynamically respond to the chronic wound healing process and achieve stage-specific dynamic modulation of the wound microenvironment (Table 1).

In this review, dynamic regulation is defined as the temporal and/or microenvironment-adaptive modulation of wound repair rather than as a general synonym for drug release. Four levels of regulation are distinguished: (i) conventional or passive delivery driven by diffusion, dissolution, swelling, or externally applied pressure; (ii) programmed release from layered, core–shell, or degradable domains according to a predetermined sequence; (iii) stimuli-responsive release triggered by pH, reactive oxygen species (ROS), temperature, radiation, enzymes, or other signals; and (iv) feedback-controlled or theranostic systems in which a sensor detects a wound-related variable and actively initiates or adjusts treatment. Mechanical closure, adhesion, and tissue stimulation constitute forms of functional regulation but should not be classified as feedback-controlled release.

**Figure 1:**
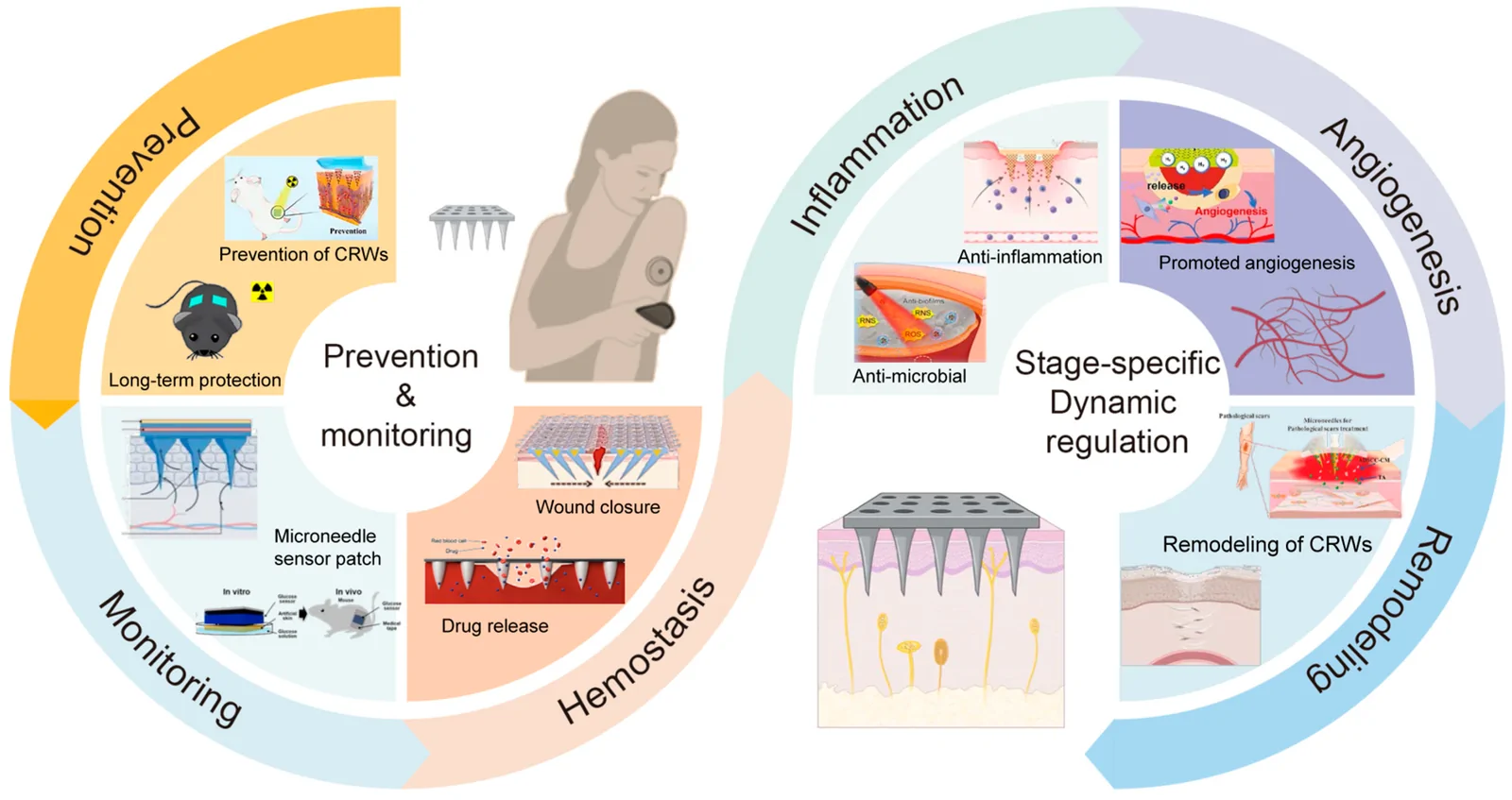
Microneedle strategies in chronic wound repair and their dynamic modulation of the wound microenvironment.

### 3.1. Hemostatic Phase

The hemostatic phase is the initial stage of wound healing. Microneedles can be useful in the hemostatic phase of a wound through both physical and pharmacological hemostasis. The most common and direct method of inhibiting bleeding is mechanical compression; therefore, hemostasis is achieved through the design of specially structured microneedles that reduce the wound area by traction or seal the wound. This stimulation of wound contraction accelerates the healing process by utilizing a wound closure strategy [74]. For example, microneedles with special bionic structures can be used for hemostasis by traction to narrow the wound area or seal the wound [45]. Zhang et al. proposed a unique claw-inspired microneedle patch with liquid metal encapsulation for accelerating incisional wound healing (Figure 2A) [75]. The patch consists of two hydrogel microneedle arrays connected by a piece of gauze. Inspired by the clamping structure of the eagle’s claw toe, which allows the bird to hold on to and grab prey, the microneedles in this patch are oriented towards each other. This design allows the structure to immobilize and tighten the wound area, preventing it from secondary dehiscence or reopening. Similarly, Deng et al. developed a microneedle patch with a lamprey teeth-inspired oriented arrangement (Figure 2B) [38]. In contrast to conventional microneedle arrays in which the needles are uniformly and vertically located on the patch, the location and size of the microneedles are intentionally altered. The central portion of the patch is covered with shorter needles, while the needles on the circular base are longer and tilted inward at 30 degrees, a structure that allows for complete binding of the skin lesion. The shorter central microneedle is inserted into the wound area, whereas the longer and marginally inclined microneedle provides a dragging force from the edge of the base to the center. Healing is accelerated by mechanical force or structural design that tightly fits the wound edges, reduces the wound area and maintains stability.

Pharmacologic hemostasis is also a common hemostatic strategy. Currently, various hemostatic agents have limited access to deep tissues and tend to be dislodged from the wound surface. In contrast, MNs have emerged as effective transdermal drug delivery systems that can overcome the resistance of the stratum corneum and penetrate the skin tissue. Yang et al. developed Yunnan Baiyao (BY)-loaded multifunctional MN patches with divisional structures for hemorrhagic wound healing (Figure 2D) [76]. BY-loaded bletilla polysaccharide dissolved rapidly and completely released in 6 min to promote platelet adhesion and activate the coagulation system, showing stronger pro-coagulability in vitro and a better hemostatic effect in a rat hepatic hemorrhage wound model. Lee et al. introduced a peripheral–topical MN patch (p-MN) designed for hemorrhagic wounds, bypassing the need for clot-piercing barriers by anchoring microneedles into peri-wound intact tissue (Figure 2C) [77]. The shelf-stable dissolving MN patch enabled sustained tranexamic acid (TXA) delivery, and its performance is dramatically enhanced in terms of bleeding time and blood loss in a heparin-treated rat model. Through the optimized design of bionic structures or the strategy of precise delivery of coagulant activators, microneedle technology provides an innovative tool to dynamically regulate the wound microenvironment in a phased manner, which is particularly suitable for early hemostasis and healing management of complex wounds.

MN technology offers complementary approaches to chronic wound management through localized delivery, physical interaction with tissue, microenvironment modulation, and sensing [78,79]. The most persuasive CRW evidence currently comes from preclinical diabetic and infected wound models. It is worth noting that studies of acute incisions, hepatic hemorrhage, oral ulcers, systemic radiation exposure, and pathological scars provide useful mechanistic or design evidence, but their performance should not be interpreted as clinical efficacy in CRWs.

### 3.2. Inflammation Regulation and Immunomodulation

Immune dysregulation plays a central role in the healing process of chronic wounds, and a persistent inflammatory response is a signature feature of chronic wounds. The pathology of CRWs is characterized by a multifactorial dynamic imbalance, in which a persistent inflammatory response is the core pathology, with an imbalance of macrophage polarization and neutrophil over-infiltration leading to the release of large amounts of pro-inflammatory factors (IL-6, TNF-α) and oxidative stress triggered by the accumulation of reactive oxygen species (ROS). Programmed cell death (e.g., necrotic apoptosis, pyroptosis) releases damage-associated molecular patterns (DAMPs, e.g., HMGB1) that further exacerbate the inflammatory cascade, forming a vicious circle of inflammation-injury.

**Figure 2:**
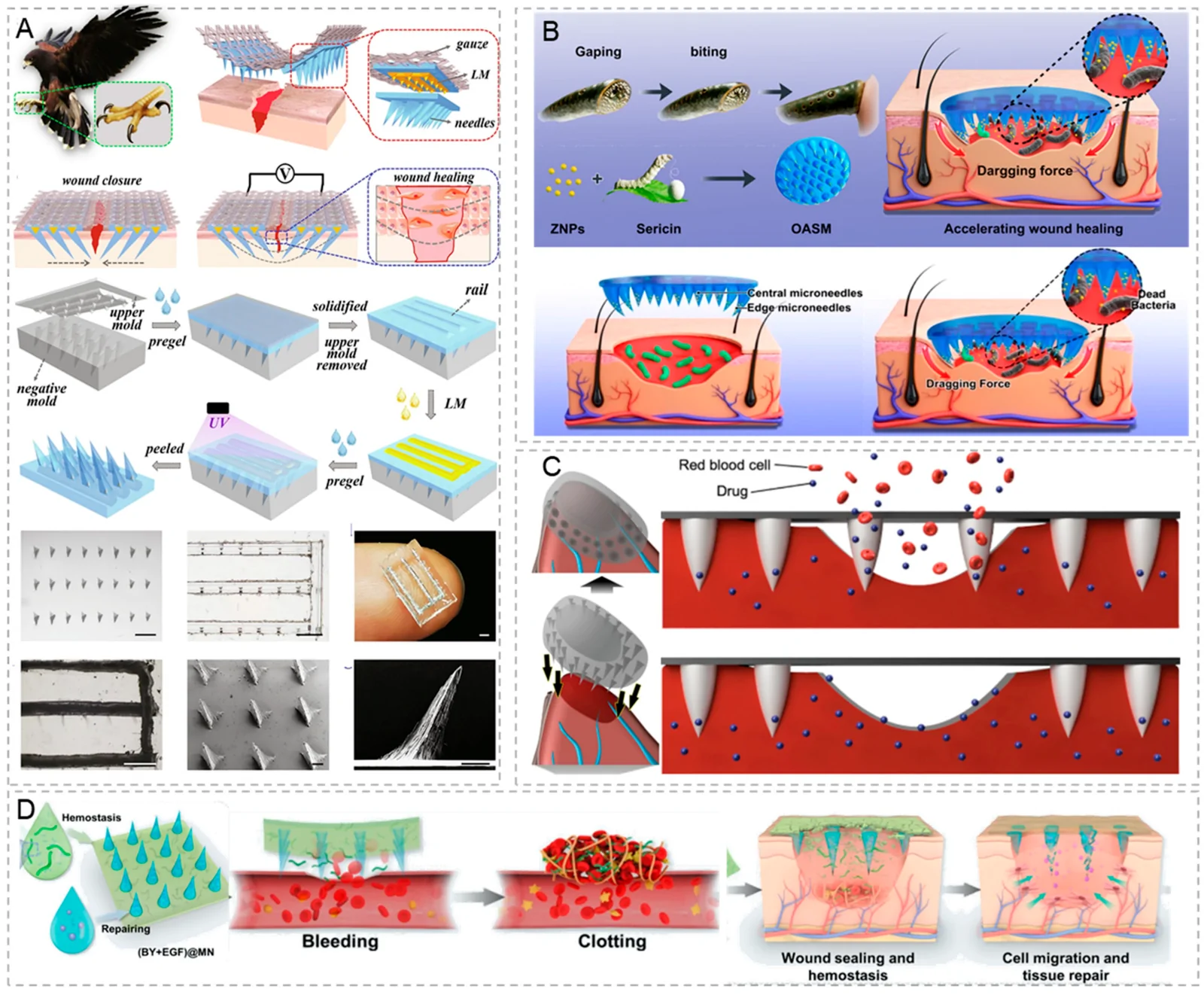
Microneedle strategies in the hemostatic phase of chronic refractory wounds. (**A**) A schematic illustration of the fabrication of claw-inspired microneedle patch with liquid metal encapsulation and its application in accelerating incisional wound healing. Adapted with permission from [75], Copyright 2021 Elsevier. (**B**) A schematic diagram illustrating of the preparation process of the microneedle patch with a lamprey teeth-inspired oriented sericin microneedle patch and its application in reducing wound area and maintaining stability. Adapted with permission from [38], Copyright 2022 American Chemical Society. (**C**) A scheme of the peripheral–topical MN patch (p-MN) designed for hemorrhagic wounds by anchoring microneedles into peri-wound intact tissue. Adapted with permission from [77], Copyright 2023 Wiley. (**D**) A schematic illustration of Yunnan Baiyao (BY)-loaded multifunctional MN patches with divisional structures for hemorrhagic wound healing. Adapted with permission from [76], Copyright 2023 Springer Nature.

Macrophage polarization from the pro-inflammatory M1 type to the anti-inflammatory M2 type is impeded in chronic wounds, and the imbalance of macrophage polarization leads to over-infiltration of neutrophils and sustained release of pro-inflammatory factors (IL-6, TNF-α). Cytokines (IL-6 and TNF-α) maintain inflammation through activation of the NF-κB pathway and impede tissue repair. Meanwhile, mitochondrial dysfunction in chronic wounds leads to the overproduction of reactive oxygen species (ROS), and the accumulation of ROS triggers oxidative stress, inhibiting fibroblast migration and angiogenesis. Microneedles are advantageous in immunomodulation, and many microneedle systems are used for this purpose [73,74,75,76,77,80,81,82,83,84]. For example, Le et al. constructed a sponge-like microneedle crosslinked with a Heparin/4-arm PEG-NH2 network, which attracted and sequestered multiple inflammatory chemokines including monocyte chemotactic protein-1 (MCP-1). Enrichment of MCP-1 on microneedles recruits and traps inflammatory monocytes within the porous structure. Subsequent removal of microneedles depletes not only the inflammatory chemokine MCP-1 but also its cellular source, resulting in accelerated diabetic wound healing due to a shortened duration of inflammation (Figure 3A) [85]. The microneedle drug system developed by Li et al. has both antibacterial and immunomodulatory functions. A nanoparticle with polydopamine (PDA)-loaded iron oxide is grafted with glucose oxidase (GOx) and hyaluronic acid (HA) and then integrated into the tips, and amine-modified mesoporous silica nanoparticles (AP-MSNs) are incorporated into the bases. By delivering microenvironment-responsive nanoparticles across the biofilm, it achieves antibacterial and immunomodulatory microenvironmental effects, which significantly promote M2 macrophage polarization and the healing of infected wounds (Figure 3B) [86].

Meanwhile, skin infections caused by bacteria, fungi, and viruses are also important factors in immune dysregulation in chronic wounds. Infections not only exacerbate tissue damage, but also lead to impeded healing processes, systemic infections, wound enlargement, increased drug resistance, and even life-threatening conditions [3,87,88,89,90]. However, traditional anti-infection methods have drawbacks. For example, topical creams and antibiotic ointments can only treat superficial infections and cannot reach deeper tissues. Hypodermic needle injection is painful and invasive. In contrast, microneedles can penetrate the biofilm barrier and release antimicrobial active drugs, leading to the destruction of the biofilm, thus exerting antimicrobial effects, and at the same time, through the targeting of inflammatory factor neutralization and macrophage polarization modulation, the dynamic regulation of reactive oxygen species (ROS) is achieved, which can alleviate the damage of oxidative stress on the traumatized cells and promote tissue repair. Yang et al. developed a microneedle bandage functionalized with dopamine-coated hybrid nanoparticles containing selenium and chlorin e6 (SeC@PA) which is capable of dual-directional regulation of reactive species (RS) generation. The MN bandage depletes endogenous glutathione (GSH) to enhance the anti-biofilm effect of RS, but also degrades GSH in biofilms through cascade reactions to generate more lethal RS for biofilm eradication. The MN bandage may be a potential self-augmented, catabolic, and dynamic therapy to effectively manage chronic diabetic wounds (Figure 3C) [91].

**Figure 3:**
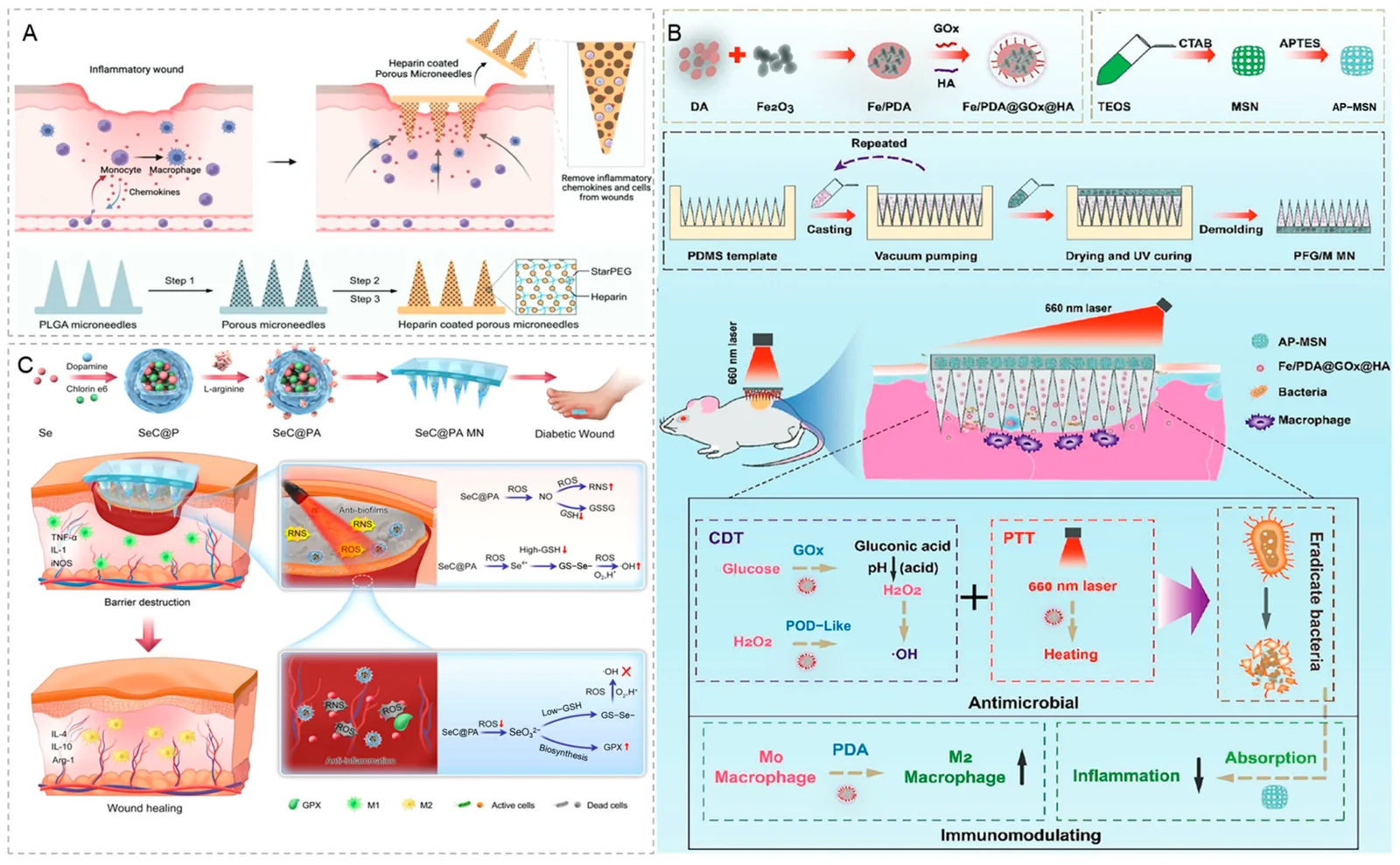
Microneedle strategies for inflammation regulation and immunomodulation of chronic refractory wounds. (**A**) A schematic illustration showing the fabrication of a sponge-like microneedle crosslinked with Heparin/4-arm PEG-NH2 and its application for anti-inflammation. Adapted with permission from [85], Copyright 2024 Wiley. (**B**) An illustration of the fabrication and principle of a microneedle drug system with both antibacterial and immunomodulatory functions. Adapted with permission from [86], Copyright 2023 Wiley. (**C**) A microneedle bandage functionalized with dopamine-coated hybrid nanoparticles for dual-directional regulation of reactive species generation. Adapted with permission from [91], Copyright 2023 Springer Nature.

### 3.3. Angiogenesis

Angiogenesis is impaired in many CRWs because reduced or dysregulated VEGF and FGF signaling, endothelial dysfunction, basement membrane thickening, ischemia, and hypoxia limit endothelial migration and vessel maturation. Layered or responsive MNs can improve local delivery and partially sequence anti-inflammatory and pro-angiogenic actions. Tao et al. designed a self-responsive H_2_-releasing microneedle patch. The microneedle continually releases hydrogen at the deep site of the diabetic wounds and achieves on-demand drug release through pH and reactive oxygen species (ROS)-responsive mechanisms. Sequential release of H_2_ and Co^2+^ significantly reduces inflammatory factors (TNF-α, IL-6), promotes angiogenesis (increased CD31 expression), and accelerates diabetic wound healing (Figure 4A) [92].

Meanwhile, material optimization enhances drug penetration, improves the local delivery efficiency of angiogenic factors, and achieves accurate delivery of angiogenic factors. Microneedles can also regulate oxidative stress, inflammation, and hypoxia of the wound through multiple mechanisms, improving the microenvironment of the wound, providing favorable conditions for angiogenesis and promoting wound re-epithelialization. Liu et al. developed a magnesium-based metal–organic framework (Mg-MOF)-loaded curcumin microneedle patch. By targeting the release of CUR and Mg^2+^, the microneedle had an excellent effect in promoting epithelial layer regeneration, optimizing connective tissue arrangement, and reducing inflammatory cell infiltration, which forms a vascular structure and has high expression of CD31 and α-SMA to accelerate oral ulcer healing (Figure 4B) [93]. Wang et al. developed polymeric microneedles based on photothermal conjugation to eliminate the biofilm and promote angiogenesis for the treatment of diabetic wounds. The photothermal effects under near-infrared light irradiation directly destroyed the biofilm and killed drug-resistant bacteria (e.g., MRSA), which regulates the angiogenesis microenvironment. The following sustained release of vascular endothelial growth factor (VEGF) effectively boosted cell migration and promoted angiogenesis to accelerate diabetic wound healing (Figure 4C) [94].

Sequential release should not be equated with synchronization to the patient’s actual healing phases. In most reported systems, timing is predetermined by layer thickness, diffusion, matrix degradation, or a single threshold such as pH or ROS. Diabetes severity, perfusion, infection, exudate composition, and inflammatory burden can shift both the threshold and the therapeutic window. Multi-analyte sensing, patient-specific calibration, adaptive thresholds, and longitudinal in vivo validation are therefore needed before phase-matched release can be claimed.

**Figure 4:**
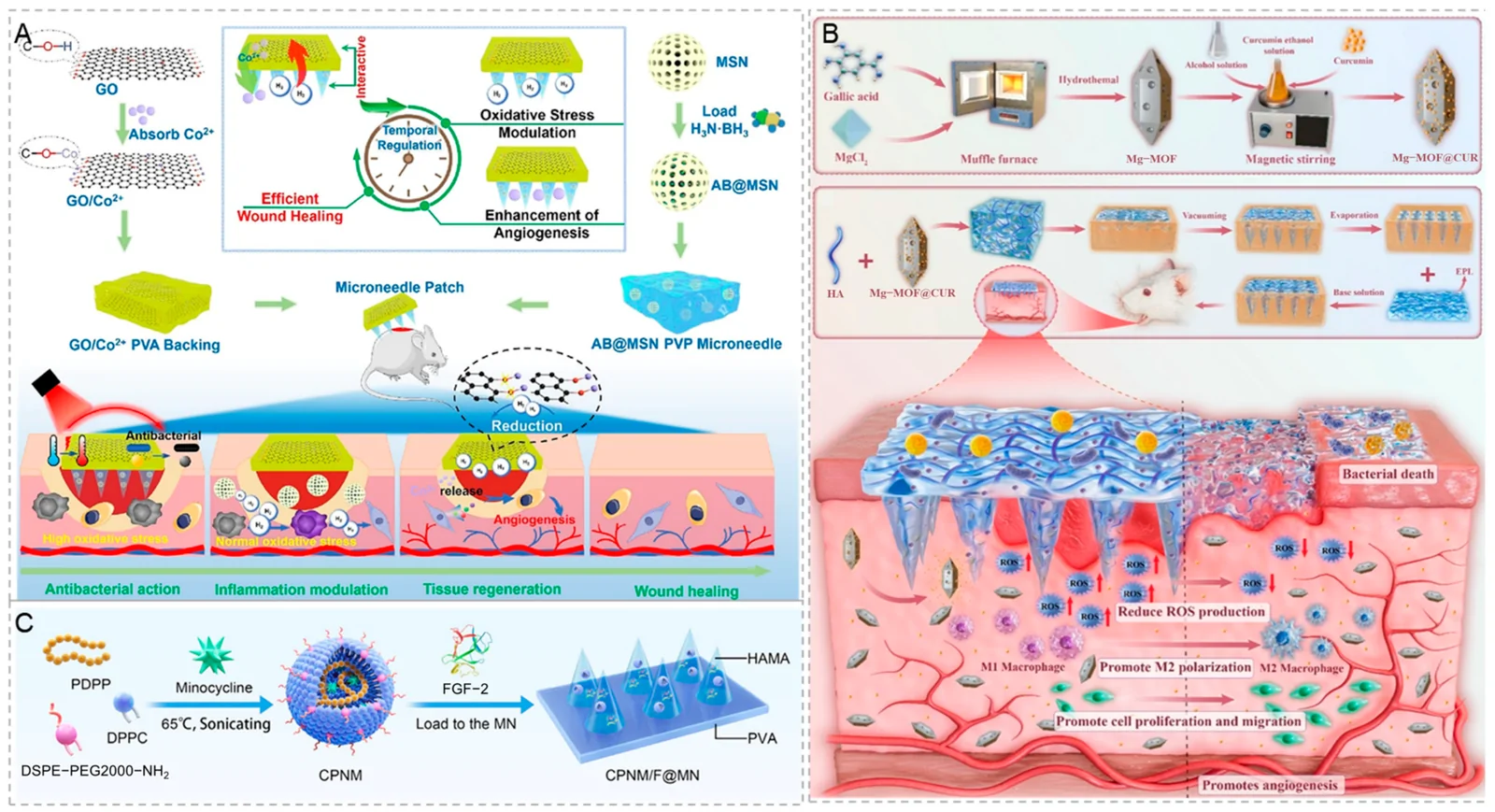
Microneedle strategies for angiogenesis in chronic refractory wounds. (**A**) A schematic diagram of a self-responsive H_2_-releasing microneedle patch that promotes angiogenesis and accelerates diabetic wound healing. Adapted with permission from [92], Copyright 2024 Elsevier. (**B**) A s scheme of a magnesium-based metal–organic framework (Mg-MOF)-loaded curcumin microneedle patch. Adapted with permission from [93], Copyright 2024 Springer Nature. (**C**) A schematic illustration showing the fabrication of polymeric microneedles based on photothermal conjugation to eliminate the biofilm and promote angiogenesis for the treatment of diabetic wounds. Adapted with permission from [94], Copyright 2025, American Chemical Society.

### 3.4. Remodeling Stage

Abnormal remodeling of the extracellular matrix is manifested by impaired senescence or phenotypic transformation of fibroblasts, reduced collagen synthesis and accelerated degradation, and an imbalance in the ratio of matrix metalloproteinases (MMPs) to tissue inhibitors (TIMPs), which makes it difficult for granulation tissue to form [9,72,73]. Dysregulated healing can also cause excessive ECM deposition, fibroblast activation, hypertrophic scarring, and tissue dysfunction. Microneedles attenuate the contraction and mechanical stresses generated by fibroblasts through physical intervention, which inhibit scar formation by creating a minimally invasive surface that initiates a normal post-inflammatory chemical cascade to stimulate fibroblast deposition and the reorientation of collagen bundles, thereby inducing collagen formation and subsequent remodeling of collagen bundles. Zhang et al. activated the YAP/TAZ signaling pathway by relying on microneedle physical stimulation, which directly inhibited scar formation through the mechanical signaling pathway (Figure 5A) [95]. Microneedle puncture induced the transformation of fibroblasts to a non-fibrotic phenotype by generating mechanical stimulation and down-regulated the expression of genes related to collagen synthesis (COL1A1, COL3A1), resulting in a reduction in scar area. However, anti-fibrotic microneedles that modulate YAP/TAZ or suppress excessive collagen deposition are most relevant to hypertrophic scars and other fibrotic remodeling disorders, not to all chronic wounds. Atrophic, ischemic, or diabetic wounds may instead require matrix support, fibroblast activity, angiogenesis, and organized collagen deposition. Broad anti-fibrotic treatment could weaken granulation tissue, delay closure, or impair tensile strength in a collagen-deficient wound [96].

**Figure 5:**
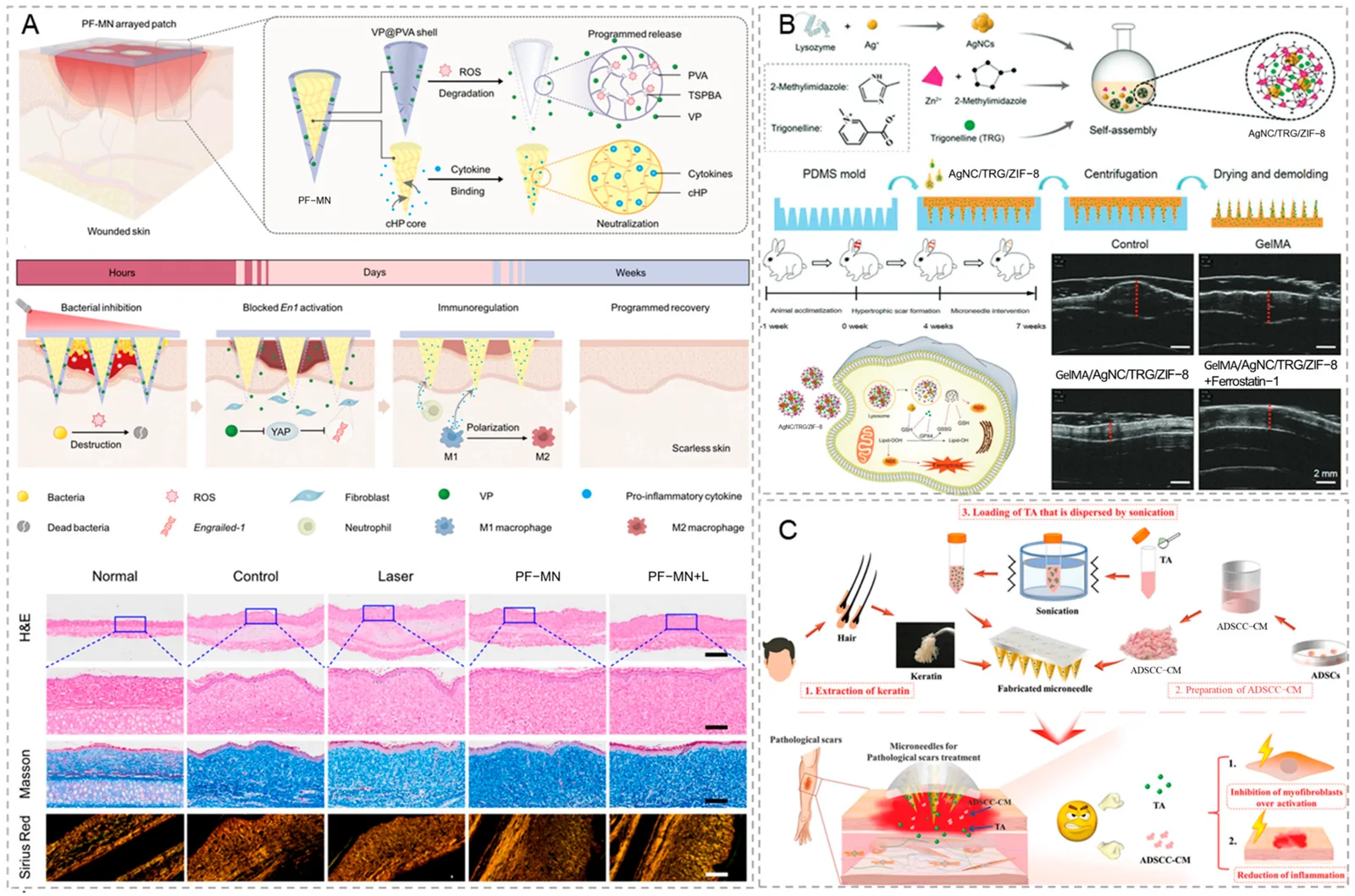
Microneedle strategies for collagen deposition in chronic refractory wounds. (**A**) A schematic diagram of core–shell microneedles inhibiting scar formation through the mechanical signaling pathway. Adapted with permission from [95], Copyright 2023 Springer Nature. (**B**) A schematic diagram of a pH-responsive microneedle showing strong antagonistic action against HS through GPX4 inactivation. Adapted with permission from [97], Copyright 2023 Wiley. (**C**) An adipose stem cell-conditioned medium-induced keratin hydrogel microneedle for pathologic scarring treatment. Adapted with permission from [98], Copyright 2024 Wiley.

Meanwhile, microneedles also serve as a drug carrier for precise and efficient drug delivery, thereby inhibiting proliferative scar formation. Zhao et al. designed a pH-responsive microneedle by encapsulating silver nanoclusters (AgNCs) and the Chinese herbal medicine trigonelline (TRG) into a zeolitic imidazolate framework-8 (ZIF-8) (Figure 4B) [97]. AgNCs have the ability to deplete GSH, while TRG inhibits the activity of glutathione peroxidase and selectively removes over-activated fibroblasts via the iron death pathway. In the ear scar model, the microneedle patch group showed strong antagonistic action against HS through GPX4 inactivation, presenting thinner muscle fibers and effectively achieving synergistic anti-scar treatment. A keratin hydrogel MN formed with adipose-derived stem-cell concentrated conditioned medium (ADSCC-CM) delivered triamcinolone acetonide (TA) and combined anti-inflammatory signaling with the regulation of myofibroblast behavior in pathological scars (Figure 4C) [98]. ADSCC-CM promotes apoptosis of dermal fibroblasts through the secretion of exosomes (miR-29a-containing), and the release of TA inhibits the TGF-β/Smad pathway. In the rabbit ear scar model, collagen deposition was significantly reduced and collagen arrangement approached that of normal skin.

**Table 1:** Comparative characteristics of the representative microneedle systems discussed in this review.

Stage	Strategy	Composition	Classification	Ref
Hemostatic phase	Bioinspired oriented antibacterial sericin microneedles	Sericin, zinc oxide nanoparticles	Infected skin wounds	[38]
	Pagoda-like multilayer microneedle	PEGDA, dodecyl-modified chitosan coating	Trauma and bleeding model	[45]
	Claw-inspired microneedle patches with liquid metal encapsulation	PEGDA, gallium–indium–tin–zinc alloy	Incisional wound	[75]
	BY-loaded multifunctional MN patches with divisional structures	Yunnan Baiyao, bletilla striata polysaccharide, epidermal growth factor (EGF), GelMA	Hemorrhagic wound	[76]
	Peripheral–topical MN patch for hemostasis	HA, TXA	Trauma and bleeding model	[77]
Inflammation regulation and immunomodulation	Microneedle photothermally encapsulated monoclonal antibodies and MXene	HA, IL-17 monoclonal antibodies, MXene	Psoriasis model	[73]
	Microneedle mediates nanosheet delivery for dual nanodynamic sterilization	PVP, black phosphorus nanosheets, copper peroxide nanodots	Infected skin wounds	[41]
	Biofilm microenvironment triggered self-enhancing photodynamic immunomodulatory	PVA, L-arginine, dopamine-coated hybrid nanoparticles containing selenium and chlorine6	Diabetic wound	[91]
	Sponge-like microneedles spatially sequester chemokines and deplete monocytes	PLGA, monocyte chemotactic protein-1, heparin and StarPEG	Psoriasis	[85]
Angiogenesis	Magnesium metal–organic framework microneedles loaded with curcumin	Hyaluronic acid, Mg-MOFs, curcumin	Oral ulcers	[93]
	Graphene oxide-integrated fish gelatin methacrylate encapsulated with bacillus peptide and VEGF	GelMA, graphene oxide, VEGF	Diabetic wound	[94]
	Self-responsive H2-releasing microneedle patch	PVA, cobalt-adsorbed graphene oxide	Diabetic wound	[92]
Collagen deposition	pH responsive self-assembly nanoplatform encapsulated silver nanoclusters and trigonelline	Gelatin methacrylate, silver nanoclusters, trigonelline encapsulated into ZIF-8	Ear scar models	[97]
	ADSCC-CM-induced keratin hydrogel microneedle patch containing triamcinolone	Triamcinolone acetonide, ADSCC-CM and keratin	Hypertrophic scar model	[98]
	Microneedles aid the transdermal delivery of shikonin	HA, Arnebiae Radix	Hypertrophic scar model	[95]
	Core–shell structured microneedle blocking Engrailed-1 (En1) activation	PVA, verteporfin, crosslinked heparin	Diabetic wound, ear scar models	[31]
	Self-powered enzyme-linked microneedle patch for scar prevention healing	Dopamine-modified pyrrole, eoliteimidazola framework, glucose oxidase and hors radish peroxidase	Diabetic wound	[96]

## 4. Monitoring and Prevention of Chronic Refractory Wounds

Chronic wounds may develop into serious complications as a result of infection, ischemia, or persistent inflammation if not prevented in time. Early risk reduction and longitudinal monitoring may therefore complement treatment. Relevant wound variables include pH, glucose, oxygenation, temperature, inflammatory mediators, microbial products, and tissue impedance, but the clinical meaning and useful sampling interval of each variable depend on wound type and patient context.

Intelligent monitoring devices (e.g., wireless bioelectronic patches) can analyze glucose, IL-6 and other biomarkers of wound exudate in real time and dynamically adjust drug release, which is of great significance in the prevention and monitoring of chronic hard-to-heal wounds [99]. Among them, microneedle technology, owing to its compact size, effective penetration capabilities, and portability, ideal for both the collection of biological data and the precise administration of drugs, has emerged as a promising technology for achieving the goals of theragnostic systems.

### 4.1. Prevention of CRWs

Functionalized MNs can achieve early protection against CRWs through cascade interventions, especially in the face of wound risk due to special circumstances such as ionizing radiation. The preventive intervention function of microneedle technology highlights its unique value. Yu et al. designed a γ-radiation-responsive microneedle system that integrates multiple sensing modules, and a flexible heater triggers granulocyte-colony-stimulating factor (G-CSF) upon detection of radiation exposure. Radiation triggers the release of G-CSF, which stimulates the bone marrow hematopoietic function, elevates the white blood cell count, and achieves on-demand administration of the drug to prevent systemic radiation syndrome (Figure 6A) [100]. Hu et al. developed a bifunctional cascade microneedle system for the prevention and treatment of RISI by co-assembling binary polyphenol natural products (e.g., tea polyphenols and anthocyanins). EGCG and catalase (CAT) form a cascade that gradually converts superoxide anion to water, which can reduce the cellular damage caused by free radicals at the early stage of radiation and play a preventive role. Meanwhile, curcumin (CUR) inhibits the inflammatory pathway, realizing the treatment of skin inflammation after radiotherapy. The nano-structural design of the microneedle system enhances skin permeability and synergizes the antioxidant effect of natural small molecules to effectively prevent and alleviate skin inflammation and oxidative damage caused by ionizing radiation (Figure 6B) [101]. Notably, Yu et al. developed amifostine-loaded armored dissolving MNs for prolonged prevention of ionizing radiation injury. The microneedle patch significantly reduces hematopoietic toxicity induced by traditional systemic administration while maintaining effective blood concentration through a localized slow-release strategy (Figure 6C) [102]. These studies support radioprotective delivery concepts, but chronic wound prevention and long-term skin outcomes require model-specific validation.

**Figure 6:**
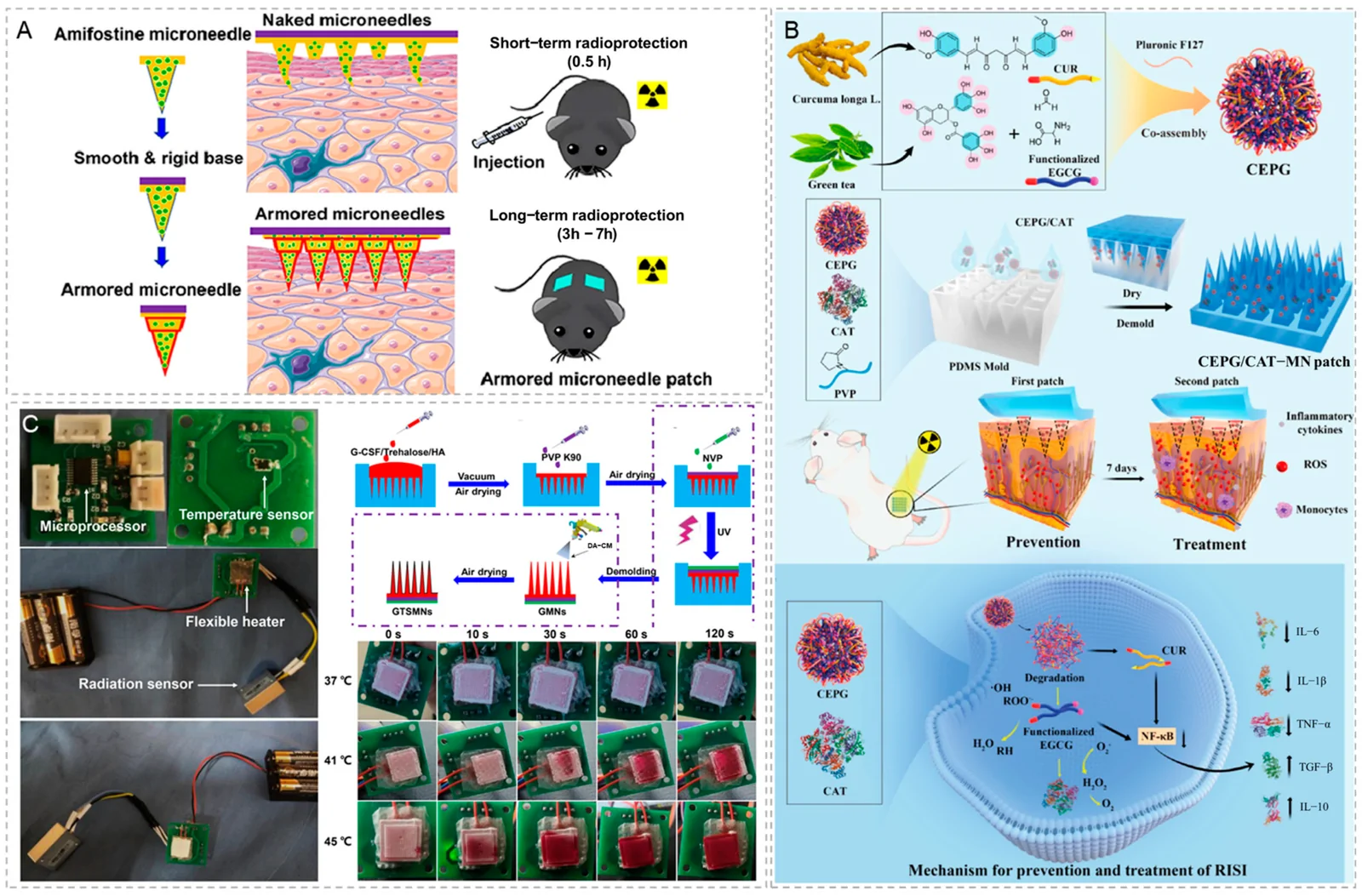
Microneedle strategies for the prevention of chronic refractory wounds. (**A**) A schematic diagram of amifostine-loaded microneedles for the long-term prevention of ionizing radiation-induced injury. Adapted with permission from [100], Copyright 2020 Elsevier. (**B**) A schematic diagram of a bifunctional cascade microneedle system for the prevention and treatment of RISI. Adapted with permission from [101], Copyright 2023 Wiley. (**C**) A schematic diagram of a microneedle system which immediately releases G-CSF into the body in response to signal feedback and provides maximal protection against ionizing radiation-induced injury. Adapted with permission from [102], Copyright 2022 Elsevier.

### 4.2. Monitoring of CRWs

The complexity of CRWs requires that clinical management take into account both dynamic monitoring and proactive intervention, and microneedle technology, with its multifunctional integration of minimally invasive penetration, real-time sensing, and precise drug delivery, is opening up new paths for the intelligent management of chronic hard-to-heal wounds [103,104]. For example, Guo et al. present a microneedle patch mimicking the structure of shark teeth for wound monitoring. By mimicking the serrated edges of shark teeth, the mechanical properties of the microneedle are optimized to reduce damage during skin insertion. At the same time, the microneedle sensor monitors biomarkers (e.g., inflammatory factors) in the wound exudate to assist in evaluating the healing process, realizing controlled release of microneedle-loaded drugs according to the wound environment (e.g., pH, temperature), and providing an integrated treatment and monitoring solution for chronic wounds (Figure 7A) [105]. This integration of bionic structure and smart materials not only improves the reliability of monitoring data, but also realizes the dynamic adaptation of treatment strategies.

The long-term stability of microneedle detection systems is also critical for chronic wound detection [106]. Kil et al. combined friction nanogenerators (TENGs) with microneedles to achieve passive energy supply by utilizing the energy generated by human mechanical movement or body fluid glucose metabolism, enabling diabetic patients to complete continuous blood glucose monitoring through a wearable device, which solves the limitation of traditional monitoring devices that rely on external power sources; the system enables wide-range continuous glucose monitoring and enhances the convenience of diabetic patient management (Figure 7B) [107]. Meanwhile, Piao et al. designed a flexible polymer microneedle patch for real-time monitoring of interstitial fluid glucose concentration through impedance changes, utilizing the chemical reaction between glucose and sensitive coatings (e.g., phenylboronic acid derivatives) to change the microneedle impedance and achieve enzyme-free detection, providing a highly stable solution for long-term, painless glucose monitoring. This enzyme-free sensing mechanism not only avoids the defect of easy inactivation of biological enzymes, but also shows excellent stability in complex wound microenvironments, providing reliable technical support for long-term monitoring (Figure 7C) [108].

**Figure 7:**
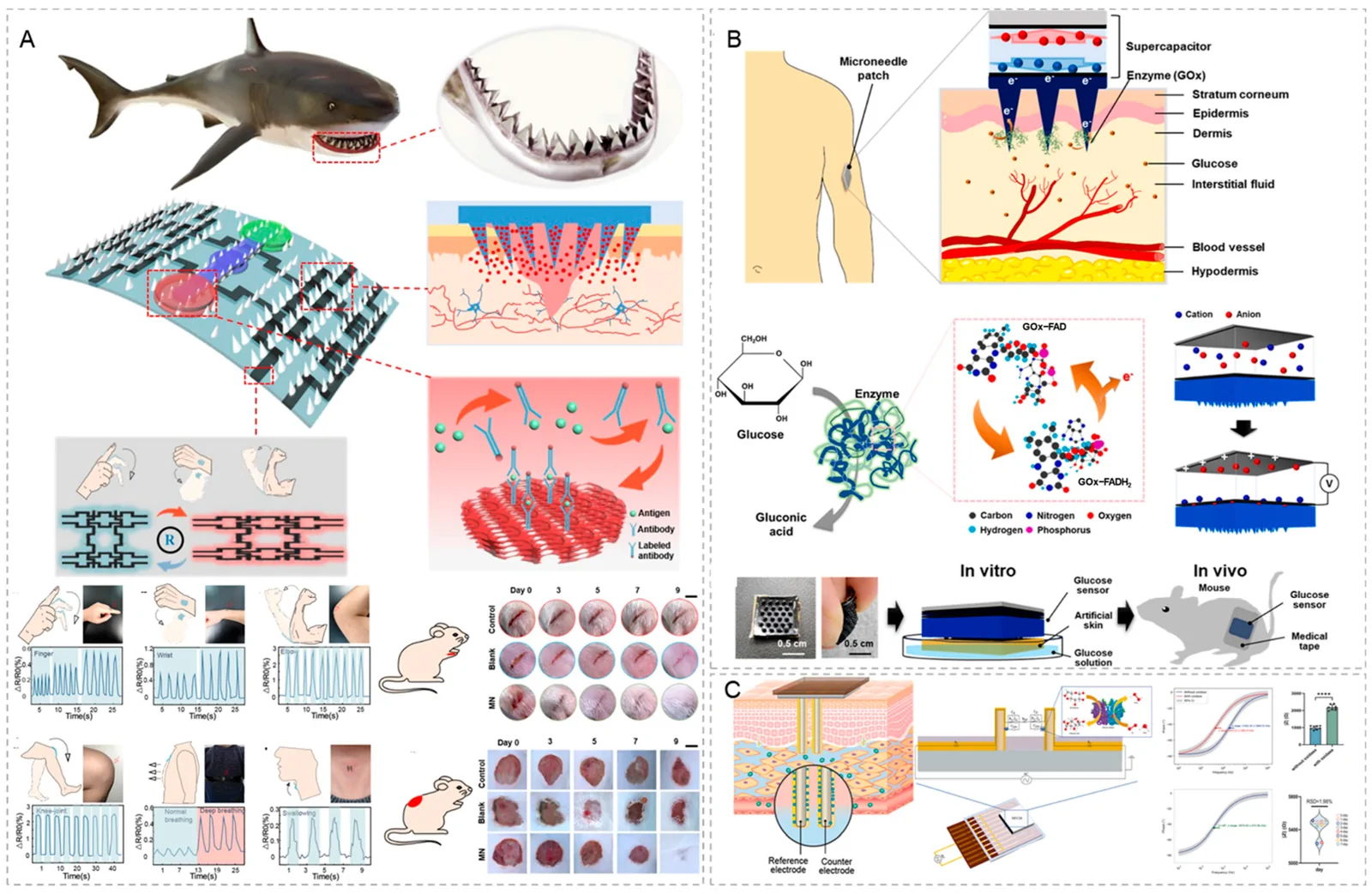
Microneedle strategies for the monitoring of chronic wounds. (**A**) A schematic diagram of a microneedle patch mimicking the structure of shark teeth for wound monitoring. Adapted with permission from [105], Copyright 2021 American Chemical Society. (**B**) A schematic diagram of a microneedle system combined with friction nanogenerators to achieve passive energy supply. Adapted with permission from [107], Copyright 2024 Elsevier. (**C**) A schematic diagram of a polymer microneedle patch for real-time monitoring of interstitial fluid glucose concentration through impedance changes. Adapted with permission from [108], Copyright 2024 Elsevier.

By optimizing bionic architectures and integrating multifunctional modules, microneedle systems have advanced beyond conventional wound management paradigms [103,104,105,106,107,108]. Some systems combine real-time monitoring with dynamic modulation of the wound microenvironment while establishing a multilayered defense framework for pre-emptive clinical interventions. From continuous glucose tracking in diabetic care to radioprotective barrier creation, and from ROS scavenging antioxidant strategies to inflammation pathway-targeted therapies, this convergence of materials engineering, biosensing technologies, and clinical pathophysiology is reshaping the landscape of chronic wound therapeutics [109,110,111,112,113,114]. The resulting platform—a synergy of wearable biocompatible devices and intelligent feedback mechanisms—may support precision management approaches for complex pathologies.

## 5. Summary and Prospects

Chronic wound management has witnessed renewed scientific interest in recent years, driven by the persistent clinical challenges of dysfunctional healing mechanisms. Although advances such as advanced dressings and negative pressure therapies have improved care paradigms, CRWs continue to confront clinicians with unmet therapeutic needs. MN technology offers complementary approaches to chronic wound management through localized delivery, physical interaction with tissue, microenvironment modulation, and sensing. Despite the remarkable advancements witnessed in preclinical research, critical barriers impede the clinical translation of microneedle-based systems, demanding urgent attention from a multidisciplinary perspective.

The microenvironment of chronic wounds is highly heterogeneous (e.g., hypoxia, high oxidative stress, biofilm formation), and chronic wound treatment often requires multi-drug combinations such as anti-infective and pro-angiogenic agents, whereas existing microneedles rely on a passive release mechanism which is unable to sense and respond to the changes in the microenvironment in real time, making it difficult to achieve the release of different drugs at different times and in different zones [8,115,116]. “Smart microneedles”, which integrate pH, temperature, and biomarker sensors, are still in the laboratory stage, and their range and signal stability still need to be optimized. Existing technologies can only realize a single signal response (e.g., pH-sensitive hydrogel microneedle) and with low sensitivity, making it difficult to capture early infection or inflammation signals, even if the external trigger is released (e.g., light, heat); its response speed is far slower than the pathological changes in the traumatic surface (e.g., ROS outbreaks in the second level), resulting in the misalignment of the therapeutic window period.

Current microneedle designs are confronted with the inherent compromise between structural integrity and controlled drug release. Soluble polymers such as gelatin and hyaluronic acid, while biocompatible, face mechanical limitations. High-strength materials (e.g., polylactic acid) ensure skin penetration but prolong degradation cycles, whereas fast-degrading carriers risk premature burst release. Meanwhile, the drug-carrying capacity of microneedles is limited; coated microneedles have been shown to have low payload capacity, necessitating repeated applications, while hollow variants exhibit exudate-dependent release patterns that are vulnerable to wound pH and enzymatic fluctuations [117,118,119,120,121,122]. These variable release characteristics undermine dose reliability, particularly for combination therapies of CRWs requiring spatiotemporal coordination.

Material and dose constraints remain important. Fast-dissolving polymers may release cargo prematurely or lack insertion strength, whereas high-strength or slowly degrading polymers such as PLA can delay release. Coated MNs have limited surface loading, dissolving MNs are constrained by needle volume, and hollow MNs may clog or show variable flow in exudative wounds [123,124,125]. Combination therapy further increases the need for quantitative loading, insertion depth, release kinetic, and pharmacokinetic data; values should be measured rather than inferred across platforms.

Long-term sensing in wound exudate is also vulnerable to biofouling [126]. Biofouling is a central limitation in wound exudate. Protein adsorption, fibrin, cells, debris, and biofilm can block pores or recognition surfaces, alter mass transport and impedance, and produce signal drift or false negative or false positive results. Enzyme-free recognition may reduce enzyme denaturation but does not prevent nonspecific fouling. Potential countermeasures include antifouling or zwitterionic coatings, size-selective membranes, reference channels, differential calibration, multiplexed readouts, scheduled patch replacement, and validation in protein- and cell-rich exudate over clinically relevant wear times. Long-term biocompatibility, degradation products, residual metals or monomers, sterilization, cold-chain requirements, and batch-to-batch manufacturing consistency must be evaluated in parallel.

Microneedle technology serves as a promising frontier in redefining CRW therapeutics. Its capacity to transcend the passive nature of conventional dressings—through active microenvironment sensing, on-demand drug orchestration, and minimally invasive delivery—positions it as a cornerstone of next-generation wound care. The potential for such innovations as stimuli-responsive biomaterials, bioinspired architectures, and modular manufacturing (e.g., 3D bioprinting) to unlock personalized, closed-loop “sense–respond–treat” frameworks is significant. Progress toward a true closed-loop “sense–respond–treat” system will require physiologically relevant chronic wound models, transparent reporting of loading and penetration, robust antifouling, scalable manufacturing, and prospective clinical studies [1,127,128]. It is anticipated that as these barriers are addressed, microneedle platforms will evolve from laboratory curiosities to indispensable clinical tools, ultimately achieving the elusive goal of predictive, preventative and personalized wound management.

## Data Availability

No new data were created or analyzed in this study. Data sharing is not applicable to this article.
